# Measuring Psychobiosocial States in Sport: Initial Validation of a Trait Measure

**DOI:** 10.1371/journal.pone.0167448

**Published:** 2016-12-01

**Authors:** Claudio Robazza, Maurizio Bertollo, Montse C. Ruiz, Laura Bortoli

**Affiliations:** 1 BIND-Behavioral Imaging and Neural Dynamics Center, Department of Medicine and Aging Sciences, “G. d’Annunzio” University of Chieti-Pescara, Chieti, Italy; 2 Department of Sport Sciences, University of Jyväskylä, Jyväskylä, Finland; University of Rome, ITALY

## Abstract

We examined the item characteristics, the factor structure, and the concurrent validity of a trait measure of psychobiosocial states. In Study 1, Italian athletes (*N* = 342, 228 men, 114 women, *M*_age_ = 23.93, *SD* = 6.64) rated the intensity, the frequency, and the perceived impact dimensions of a psychobiosocial states scale, trait version (PBS-ST), which is composed of 20 items (10 functional and 10 dysfunctional) referring to how they usually felt before an important competition. In Study 2, the scale was cross validated in an independent sample (*N* = 251, 181 men, 70 women, *M*_age_ = 24.35, *SD* = 7.25). The concurrent validity of the PBS-ST scale scores were also examined in comparison with two sport-specific emotion-related measures and a general measure of affect. Exploratory structural equation modeling and confirmatory factor analysis of the data of Study 1 showed that a 2-factor, 15-item solution of the PBS-ST scale (8 functional items and 7 dysfunctional items) reached satisfactory fit indices for the three dimensions (i.e., intensity, frequency, and perceived impact). Results of Study 2 provided evidence of substantial measurement and structural invariance of all dimensions across samples. The low association of the PBS-ST scale with other measures suggests that the scale taps unique constructs. Findings of the two studies offer initial validity evidence for a sport-specific tool to measure psychobiosocial states.

## Introduction

The beneficial and detrimental effects of emotions on performance have been widely investigated in sport psychology [1–4]. As a result of this interest, researchers have developed a number of instruments to assess competitive anxiety [5, 6] or other emotions [7]. Ruiz, Hanin, and Robazza [8] have recently proposed an individualized profiling procedure to assess a large array of athletes’ performance-related experiences termed psychobiosocial states. Grounded in the individual zones of optimal functioning (IZOF) model [2, 9, 10], the profiling procedure enables researchers and practitioners to identify and assess the athletes’ idiosyncratic descriptors of experiences surrounding successful and unsuccessful performances [11]. This comprehensive assessment of the athlete’s functional and dysfunctional psychobiosocial states includes *affective*, *cognitive*, *motivational*, *volitional* (psychological), *bodily-somatic*, *motor-behavioral* (biological), *operational*, *and communicative* (social) modalities. In addition to in-depth individual profiles, researchers can benefit from a standardized tool in the assessment of athletes’ states, particularly with large samples of participants. Therefore, the purpose of our study was to examine the item characteristics and the factor structure of the items comprised in the individualized profiling of psychobiosocial states [8]. Another objective was to investigate the concurrent validity of the tool in comparison with other instruments.

The conceptualization of psychobiosocial states in the IZOF model is to some extent akin to other theoretical perspectives used to investigate emotions in achievement settings. For example, the control-value theory [12] provides an integrative approach for studying a variety of emotions experienced in different contexts, including academic settings, sport, and professions. Emotions are construed as sets of interrelated psychological processes, in which affective, cognitive, motivational, and physiological components are fundamental. Anxiety, for instance, can involve affective (feeling uncomfortable), cognitive (worry), motivational (withdrawal tendencies), and physiological (peripheral activation) components [13, 14]. Another leading perspective in achievement contexts is the biopsychosocial model of challenge and threat, so named because it integrates biological, psychological, and social levels of analysis to explain motivational processes of human performance [15, 16]. The biopsychosocial model accounts for the autonomic and endocrine influences on the cardiovascular system (the biological level), the cognitive and affective influences on evaluative processes (the psychological level), and the interplay among intraindividual, interindividual, and environmental aspects (the social psychological level). Challenge and threat motivations are embedded in these levels of analysis and their interplay. In this view, challenge and threat are motivational states evoked in the interaction between the person and the situation, with affective, cognitive, and physiological antecedents and consequences.

Rooted in the IZOF model [9, 17], psychobiosocial states have been assessed in a number of studies conducted in the sport context [11, 18–21] and in the physical education setting [22–27]. Previous assessments were typically conducted using a list containing an earlier version of functional and dysfunctional descriptors targeting the psychobiosocial components (modalities) of the individual’s achievement experience. Each item represented a discrete state and was composed of two or three descriptors to transmit a clear representation of one’s experience related to sport or physical education. In some studies, for example, functional and dysfunctional items for each modality were as follows: “happy” and “depressed” (affective modality); “certain” and “doubtful” (cognitive); “committed” and “disengaged” (motivational); “physically fresh” and “stiff muscles” (bodily-somatic); “active” and “clumsy” (motor-behavioral); “proficient” and “ineffective” (operational); “collaborative” and “lonely” (communicational). In a trait-like assessment, participants rated each item on a 5-point intensity scale thinking of how they usually felt within their sport or physical education context. In two previous studies on elite basketball players [28] and carom billiards players [29] a state-like assessment was implemented within 1 hr prior to competition.

Taken together, findings of the studies mentioned above show that the assessment of psychobiosocial states contributes to our understanding of relevant research questions in sport and physical education. For example, biological markers of precompetitive anxiety and activation (i.e., testosterone, cortisol, α-amylase, and chromogranin A) correlated with functional states of basketball players [28]. Furthermore, functional states mediated the effect of technical and cognitive self-efficacy on performance of carom billiards players [29]. In the physical education context [24], results showed that achievement motivational climates induced by teachers tend to determine in their students states consistent with the motivational atmosphere, thus supporting the advantages of a task-involving climate to enhance pleasant emotional experiences. The research benefits deriving from assessing psychobiosocial states highlight the need of a reliable and valid measure. Actually, Bortoli and Robazza [22] examined the factor structure of the initial version of the psychobiosocial states scale in a large sample of 11 to 14-year-old physical education students. They showed a 2-factor solution (i.e., functional and dysfunctional dimensions) to be acceptable and reliable. The same 2-factor solution was found in an Italian sample of young athletes aged 13–14 years [19]. However, the *volitional* modality of psychobiosocial states was not considered in these initial measures, and conceptualized later on [10]. Moreover, data on the factor structure and the concurrent validity of the scale for athletes have been missing.

Another limitation in earlier studies, with the exception of Robazza et al.’s [28] investigation, relies in the assessment of athletes’ descriptors only in terms of intensity. Several anxiety scholars have claimed that the *intensity* of anxiety symptoms is one aspect of the anxiety response. Another main aspect to consider is the individual’s perception of facilitative or debilitative effects on performance [30, 31]. Thus, researchers have recommended including a *functional impact* (also called *direction*) scale to take into account the perceived impact on performance of anxiety symptoms [30, 31]. Together with the intensity and perceived impact dimensions, researchers have also advocated the inclusion of a *frequency* scale in anxiety measures, because of the distinct patterns of intensity and frequency of anxiety symptoms found in the precompetition period [32–34]. According to this call, intensity, frequency, and perceived impact dimensions were incorporated in the Competitive State Anxiety Inventory-2 [5] for the assessment of cognitive and somatic symptoms of anxiety and self-confidence [35, 36].

Based on the above review of previous research, the purposes of the present investigation were twofold. In Study 1, we evaluated the construct validity and reliability of the intensity, frequency, and perceived impact dimension scores of a trait-like version of the psychobiosocial states scale (from now on referred to as the PBS-ST scale). In Study 2, we examined the invariance of the PBS-ST scale across samples, gender, and type of sport (individual vs. team). We also assessed the concurrent validity of the PBS-ST scale in comparison with other emotion and affect related measures. According to Messick [37] and Martinent, Guillet-Descas, and Moiret [38], construct validation involves at least substantive, structural, and external aspects. The substantive aspect refers to the theoretical rationale that delineates the construct under investigation. The structural aspect relates to providing evidence of factorial validity and reliability of the construct of interest. The external aspect refers to whether the investigated construct is related to other variables consistent with a theoretical foundation. In Study 1 and Study 2 we examined the structural and external aspects respectively, given that the theoretical rationale of the scale has been addressed in previous research [8].

## Method

### Study 1

Study 1 was conducted to examine the item characteristics, the factor structure, and the reliability of the PBS-ST scale.

#### Participants

Participants were 342 athletes (228 men, 114 women), aged 16 to 50 years (*M* = 23.93, *SD* = 6.64), drawn from main sport clubs located in central Italy. The study took place from February to April 2014. Two researchers approached the athletes in their sporting field or gym before regular practice sessions. The athletes’ rate of drop-out was about 5%. The athletes who voluntarily agreed to take part in the study had between one and 30 years of competitive experience (*M* = 11.50, *SD* = 5.85) and represented a range of individual sports (*n* = 146; e.g., tennis, swimming, track & field, martial arts, gymnastics, cycling, fencing) and team sports (*n* = 196; e.g., soccer, volleyball, basketball, rugby, water polo, baseball, softball, futsal). Athletes competed at regional level (83%), national level (12%), and international level (5%). No significant differences were found for age and sport experience between men and women or individual and team sports (*p* > .05).

#### Measure

The PBS-ST scale consists of 20 rows of 80 adjectives (from 3 to 6 per row) to assess eight state modalities (i.e., affective, cognitive, motivational, volitional, bodily-somatic, motor-behavioral, operational, and communicative). The Italian version of the PBS-ST was developed from the original English version of the Individualized Profiling of Psychobiosocial States [8] (see S1 Appendix). The English version contains 20 items of 74 adjectives (3–4 per row to form an item) targeting the 8 functional (+) and dysfunctional (-) modalities of a psychobiosocial state. In particular, the Affective modality is assessed by six rows of adjectives for pleasant/functional affect (+), pleasant/dysfunctional affect (-), unpleasant/functional anxiety (+), unpleasant/dysfunctional anxiety (-), functional anger (+), and dysfunctional anger (-). For the other seven modalities two rows of synonym adjectives assessed functional or dysfunctional states. The intensity response dimension of each item was rated on a 5-point Likert scale ranging from 0 (*not at all*) to 4 (*very*, *very much*). The frequency dimension was also rated on a 5-point Likert scale anchored by 0 (*never*) and 4 (*almost always*) referring to the hour prior to competition. Finally, the perceived impact dimension was rated on a bipolar 7-point Likert scale ranging from −3 (*very harmful*) to +3 (*very helpful*), according to the individual’s perception of functional or dysfunctional effects on performance, with 0 indicating no effect. The scale was translated into Italian using the backward translation technique. Three Italian researchers fluent in English independently translated the scale from English to Italian. The researchers discussed their translations until they reached agreement on all adjectives. The translated scale was then retranslated into English by a native English speaker. The researchers checked the translated and retranslated texts to make sure they reflected the original meaning. Finally, consensus was reached on the Italian version of the scale.

#### Procedure

Following approval from the ethics committee for biomedical research of the University of Chieti-Pescara, and according to the declaration of Helsinki, we contacted sport managers and coaches and explained the general purpose of the investigation to obtain authorization to approach the athletes. Prior to scale administration, participants were informed about the general purpose of the study and presented with instructions indicating that there were no right or wrong answers. They also received instructions designed to minimize social desirability bias, and emphasis was placed on the confidentiality of responses. Written informed consent approved by the ethics committee was obtained from participants or parents in the case of participants under 18 years of age. Athletes completed the PBS-ST scale individually in a quiet room before regular practice sessions. An investigator administered the scale to groups of up to five participants who voluntary took part in the study. Athletes were instructed to respond to the PBS-ST scale items referring to how they usually feel within the hour before an important competition. In particular, athletes were requested to choose one descriptor from each row that best reflected their experiences. Then, they were asked to score each descriptor in regards to its intensity, frequency, and perceived impact on performance.

#### Data Analysis

We calculated the frequency of descriptors the athletes chose across each modality to identify the most- and least-often selected adjectives. Descriptive statistics, Pearson product-moment correlation coefficients, reliability alpha values, and composite reliability values of the latent variables were also computed.

We examined the factorial validity of PBS-ST scale, with items rated in intensity, frequency, and perceived impact dimensions, using Exploratory Structural Equation Modeling (ESEM) [39], and Confirmatory Factor Analysis (CFA). ESEM is a strategy that allows for the integration of exploratory and confirmatory factor analysis within the same solution [40]. Unlike standard CFA, where cross-loadings are constraint to zero, in ESEM all factor loadings and cross loadings are estimated, while some factors can be specified within a given measurement model. In addition, factor loading matrices can be rotated (we used a Bi-Geomin rotation in our analysis). ESEM models were estimated using the robust maximum likelihood estimator (MLR), while CFA models were estimated using the maximum likelihood parameter estimates (MLM) with standard errors and a mean-adjusted chi-square test statistic that is robust to non-normality [41]. According to Byrne [42], the MLM estimator is most appropriately used with continuous data non-normally distributed and complete. All data analyses were performed in Mplus version 7.31 [41].

Following the suggestions of several researchers [43, 44], different indices were chosen to assess model fit: chi-square (*χ*^2^), normed chi-square (*χ*^2^/*df*), comparative fit index (CFI), Tucker Lewis fit index (TLI), root mean square error of approximation (RMSEA), and standardized root mean square residual (SRMR). Values for CFI and TLI greater than .90, and RMSEA and SRMR lower than .08, are considered evidence of acceptable fit [45], while values for CFI and TLI close to .95, and RMSEA and SRMR lower than .05, are evidence of good fit [43]. Furthermore, a *χ*^2^/df value less than 5 indicates an acceptable model fit [46]. Akaike’s Information Criterion (AIC) values were included as a measure to compare the fit of alternative models. Improvements in model fits are reflected in higher values of CFI and TLI, and lower values of AIC, *χ*^2^, *χ*^2^/df, RMSEA, and SRMR.

#### Results

Descriptive statistics showed that all adjectives were selected by participants. The 10 most chosen descriptors were: focused [Cognitive(+), 64.62%], motivated [Motivational(+), 64.62%], physically-charged [Bodily-somatic(+), 64.04%], worried [Anxiety(-), 61.40%], unmotivated [Motivational(-), 57.31%], nervous [Anxiety(+) 57.31%], uncommunicative [Communicative(-), 52.34%], sluggish movement [Motor-behavioral(-), 48.54%], overjoyed [Pleasant affective(-), 47.08%], and fighting spirit [Anger(+), 44.74%]. The 10 least selected descriptors were: persistent [Volitional(+), 8.77%], purposeful [Volitional(+), 7.89%], apprehensive [Anxiety(-), 7.89%], undetermined [Volitional(-), 7.31%], carefree [Affective(+), 6.73%], discontented [Affective(+), 6.43%], annoyed [Anger(-), 6.14%], physically exhausted [Bodily-somatic(-), 5.26%], resentful [Anger(-), 5.26%], and joyful [Affective(+), 3.22%]. Descriptive statistics of items are reported in Table 1.

**Table 1:** Descriptive statistics of intensity, frequency, and perceived impact dimensions of psychobiosocial states from Study 1 (*N* = 340). Note: (+) = item categorized as functional; (-) = item categorized as dysfunctional. *M* = mean, *SD* = standard deviation, SK = skewness, K = kurtosis.

Modality	Intensity				Frequency				Perceived Impact			
	*M*	*SD*	SK	K	*M*	*SD*	SK	K	*M*	*SD*	SK	K
**Pleasant affective(+)**	2.47	0.91	-0.17	-0.08	2.74	0.95	-0.74	0.49	1.16	1.39	-0.62	-0.28
**Anxiety(+)**	1.47	1.07	0.50	-0.29	1.81	1.14	-0.04	-0.87	-0.73	1.41	0.37	-0.37
**Anger(+)**	2.79	0.92	-0.42	-0.49	2.78	0.99	-0.38	-0.67	1.88	1.17	-1.45	2.33
**Cognitive(+)**	2.82	0.94	-0.37	-0.58	2.94	0.87	-0.83	1.02	2.04	1.13	-1.62	3.01
**Motivational(+)**	3.04	0.89	-0.70	0.04	2.99	0.92	-0.82	0.48	1.76	1.31	-1.33	1.63
**Volitional(+)**	2.94	0.86	-0.42	-0.54	2.93	0.81	-0.54	0.13	1.95	1.15	-1.39	2.09
**Bodily-somatic(+)**	2.86	0.93	-0.40	-0.52	2.88	0.84	-0.43	-0.21	1.95	1.10	-1.47	2.49
**Motor-behavioral(+)**	2.37	0.94	-0.29	-0.06	2.47	1.01	-0.44	-0.13	1.53	1.25	-0.63	-0.39
**Operational(+)**	2.40	0.97	-0.27	-0.03	2.58	0.88	-0.74	0.79	1.55	1.29	-1.05	1.14
**Communicative(+)**	2.41	1.21	-0.36	-0.75	2.47	1.16	-0.56	-0.40	1.25	1.27	-0.35	-0.33
**Pleasant affective(-)**	2.29	1.05	-0.17	-0.36	2.41	1.03	-0.50	0.01	1.19	1.35	-0.70	0.30
**Anxiety(-)**	1.49	1.00	0.56	-0.11	1.82	0.99	0.10	-0.28	-0.59	1.60	0.39	-0.47
**Anger(-)**	1.12	1.06	0.82	0.12	1.26	1.07	0.56	-0.37	-0.47	1.62	0.36	-0.42
**Cognitive(-)**	1.09	0.96	0.85	0.43	1.51	1.00	0.19	-0.44	-0.99	1.49	0.51	-0.19
**Motivational(-)**	0.58	0.73	1.36	2.24	0.93	0.97	0.95	0.47	-1.19	1.57	0.72	-0.03
**Volitional(-)**	0.95	0.92	1.11	1.30	1.25	1.07	0.53	-0.28	-1.08	1.63	0.70	-0.02
**Bodily-somatic(-)**	1.62	1.00	0.38	-0.29	1.93	0.95	-0.05	-0.22	-0.74	1.49	0.30	-0.68
**Motor-behavioral(-)**	0.81	0.83	1.27	2.30	1.22	0.98	0.57	-0.01	-1.17	1.52	0.76	0.22
**Operational(-)**	1.01	0.86	0.80	0.53	1.36	0.97	0.37	-0.27	-1.13	1.48	0.67	-0.06
**Communicative(-)**	1.37	1.24	0.53	-0.79	1.73	1.36	0.19	-1.19	0.39	1.44	-0.19	0.26

Before factor analysis, intensity, frequency, and perceived impact data scores were screened for missing values, skewness, kurtosis, and multivariate outliers. No missing data values were found. Two multivariate outliers were detected using Mahalanobis’ distance (*p* < .001) and then removed from data set and subsequent analyses. Because the assumption of multivariate normal data distributions was violated, ESEM and CFA models were estimated using MLR and MLM, respectively.

Three 2-factor models were tested through ESEM to assess independently the tenability of the intensity, frequency, and perceived impact dimensions. The three models included 20, 16, and 15 items, in which problematic items were progressively discarded. The decision to remove an item was based on a high value of modification indices (> 15) or a low factor loading (< .40) on the hypothesized latent factor. The final 15-item solution was also assessed using CFA, which is a more restrictive analysis than ESEM. This 2-factor, 15-item solution was also compared to an alternative 1-factor model in which all items loaded on a single factor.

Factor analysis results are summarized in Table 2. The 20-item scale assessing the intensity, frequency, and perceived impact dimensions did not provide acceptable fit. Items of the Anxiety(+) and Pleasant affective(-) modalities did not load onto the expected factor. As it can be seen from the mean scores of perceived impact (Table 1), the Anxiety(+) modality, measured by “nervous, restless, discontented, dissatisfied” descriptors, was actually perceived as dysfunctional rather than functional. Similarly, the Pleasant(-) modality, represented by “overjoyed, complacent, pleased, satisfied”, was experienced as functional. Therefore, these two modalities were discarded from following analyses. Furthermore, loadings of both Communicative(+) and Communicative(-) modalities were below the cut-off criterion of .40, and thus removed from further analysis. Although the fit of the 16-item scale improved (see Table 2), inspection of modification indices suggested that adding a path between the functional latent factor and the Anger(-) modality would substantially enhance the model fit. Rather than adding this path, we decided to exclude the Anger(-) modality in order to attain a clear distinction between functional and dysfunctional factors.

**Table 2:** Fit indices for the 2-factor models of the intensity, frequency, and perceived impact dimensions of the PBS-ST scale from Study 1. Note: ESEM = Exploratory Structural Equation Modeling, CFA = Confirmatory Factor Analysis, *χ*^2^(*df*) = chi-square (degrees of freedom), CFI = comparative fit index, TLI = Tucker Lewis fit index, RMSEA = root mean square error of approximation, SRMR = standardized root mean square residual, AIC = Akaike’s Information Criterion. CFA on the 15 items unidimensional model of the perceived impact dimension did not converge.

Dimension	Model	*χ*^2^(*df*)	*χ*^2^/*df*	CFI	TLI	RMSEA (90% CI)	SRMR	AIC
**Intensity**	20 items (ESEM)	293.190 (151)	1.942	.878	.847	.053 (.044–.062)	.048	17605.797
	16 items (ESEM)	148.557 (89)	1.669	.935	.913	.044 (.031–.057)	.038	13548.879
	15 items (ESEM)	114.557 (76)	1.507	.954	.936	.039 (.023–.053)	.035	12617.145
	15 items (CFA)	131.570 (89)	1.478	.950	.942	.038 (.023–.051)	.048	12613.213
	15 items unidimensional (CFA)	447.398 (90)	4.971	.584	.515	.108 (.098–.118)	.121	12969.401
**Frequency**	20 items (ESEM)	259.451 (151)	1.718	.898	.872	.046 (.036–.055)	.049	18191.031
	16 items (ESEM)	130.925 (89)	1.471	.944	.922	.038 (.016–.050)	.038	14035.493
	15 items (ESEM)	104.335 (76)	1.373	.963	.949	.033 (.015–.048)	.036	13083.651
	15 items (CFA)	129.687 (89)	1.457	.949	.940	.037 (.022–.050)	.051	13090.190
	15 items unidimensional (CFA)	432.627 (90)	4.807	.571	.500	.106 (.096–.116)	.114	13436.678
**Perceived impact**	20 items (ESEM)	262.513 (151)	1.738	.898	.872	.047 (.037–.056)	.045	22464.694
	16 items (ESEM)	179.658 (89)	2.019	.899	.864	.057 (.045–.070)	.047	17932.796
	15 items (ESEM)	155.124 (76)	2.041	.906	.870	.055 (.043–.068)	.045	16688.956
	15 items (CFA)	169.643 (89)	1.906	.908	.891	.052 (.040–.063)	.056	16686.875

**Fig 1:**
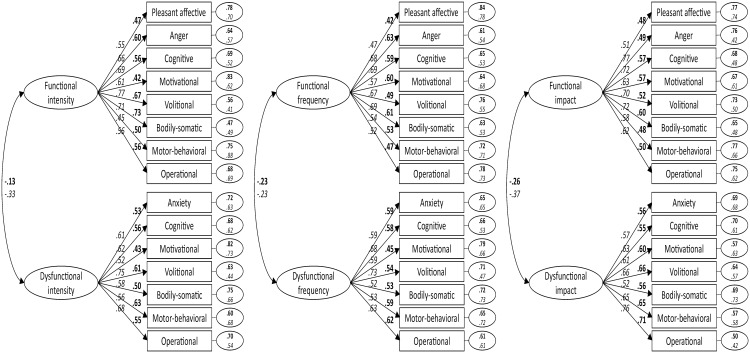
Standardized factor loadings, error variances, and correlations between latent constructs of the 15-item PBS-ST scale from Study 1 (bold; *N* = 340) and Study 2 (italic; *N* = 249) derived from confirmatory factor analysis. All factor loadings are significant at *p* < .001 (two-tailed).

The final 15-item scale reached acceptable fit indices on both ESEM and CFA. The Satorra-Bentler scaled chi-square difference test (intensity, ΔS-B *χ*^2^ = 33.01, Δ*df* = 13, *p* = .002; frequency, ΔS-B *χ*^2^ = 26.76, Δ*df* = 13, *p* = .013; direction, ΔS-B *χ*^2^ = 24.48, Δ*df* = 13, *p* = .027) and the AIC values provided evidence of fit superiority of the 15-item scale compared to the 16-item scale. The 15-item model was compared to an alternative model in which all items loaded on a single factor (i.e., 15 items, unidimensional model). As expected, the unidimensional model showed a poor fit to the data. Fig 1 displays standardized factor loadings, error variances, and correlations between latent constructs (i.e., functional and dysfunctional psychobiosocial states) of the 15-item PBS-ST scale. All factor loadings were significant at *p* < .001 (two-tailed). Table 3 contains the means, standard deviations, Pearson-product moment correlation matrix, reliability alpha values, and composite reliability values for the latent variables.

**Table 3:** Means, standard deviations, and Pearson-product moment correlation matrix for the latent variables from Study 1. Note: Cronbach’s alphas (left side) and composite reliability values (right side) appear in the diagonal.

Latent variables	(1)	(2)	(3)	(4)	(5)	(6)
**(1) Functional intensity**	(.788, .790)					
**(2) Dysfunctional intensity**	-.119	(.745, .748)				
**(3) Functional frequency**	.828	-.116	(.765, .768)			
**(4) Dysfunctional frequency**	-.207	.957	-.236	(.761, .762)		
**(5) Functional perceived impact**	.590	-.204	.611	-.237	(.752, .754)	
**(6) Dysfunctional perceived impact**	.075	-.079	-.047	-.001	-.264	(.806, .807)
**Means**	2.71	1.08	2.79	1.43	1.73	-0.98
**Standard deviations**	0.58	0.57	0.56	0.64	0.74	1.05

### Study 2

The purpose of Study 2 was to cross validate in a second independent sample the 2-factor, 15-item solution of the PBS-ST scale found in Study 1, and to examine possible gender and sport (individual vs. team) differences in the responses. An additional objective was to explore concurrent validity through correlations with two other sport-specific emotion-related measures and a general measure of affect often used in sport psychology research.

#### Participants

We involved a voluntary sample of 251 athletes (181 men, 70 women), aged 16 to 52 years (*M* = 24.35, *SD* = 7.25), from sport clubs in central Italy. The study took place from February to April 2015. The rate of drop-out was around 4%. The athletes had between 1 and 32 years of sport experience (*M* = 10.61, *SD* = 6.04) and practiced individual sports (*n* = 125) and team sports (*n* = 135) at regional level (80%), national level (15%), and international level (5%). No significant differences were shown for age and sport experience between men and women or individual and team sports (*p* > .05).

### Measures

#### The PBS-ST scale

We used the 2-factor, 15-item solution of the PBS-ST scale found in Study 1 (see S1 Appendix) to assess the intensity, frequency, and perceived impact of functional and dysfunctional states.

#### The Sport Emotion Questionnaire (SEQ)

The SEQ [7] is a 22-item, sport-specific measure of precompetitive emotion derived from the experience of athletes. Based on a categorical perspective of emotions, the SEQ assesses intensity of anger (e.g., annoyed, irritated), anxiety (e.g., nervous, apprehensive), dejection (e.g., unhappy, disappointed), excitement (e.g., enthusiastic, energetic), and happiness (e.g., joyful, cheerful). Jones et al. [7] reported CFA values indicating that the 5-factor structure provided acceptable model fit. In a recent study [47], sport performers were required to rate how they had felt in their organizational environment during the past month. The results supported the validity and reliability of the SEQ, suggesting that the measure is conceptually well designed and relevant to sport performers. The scale was translated into Italian using the backward translation technique previously described (see the Measure section in Study 1). In our study, the question “how you feel right now, at this moment, in relation to the upcoming competition” [7] was modified asking the athletes to refer to how they usually feel before an important competition. This change was made to align directions to the PBS-ST scale instructions. Alike the PBS-ST scale and previous research using the SEQ [48], we also included the Likert scales for intensity, frequency, and perceived impact dimension ratings.

#### The Positive and Negative Affect Schedule (PANAS)

Grounded in a dimensional approach of affective states, Watson, Clark, and Tellegen [49] developed the PANAS. The scale is a general measure of affect comprising two 10-item adjective checklist subscales named positive affect (e.g., enthusiastic, determined, active) and negative affect (e.g., afraid, distressed, hostile). The PANAS has been often applied in sport psychology to assess affect intensity [50–52]. Nicolas et al. [51] provided validity and reliability evidence of the PANAS measuring intensity of affect and perceived impact of affect on performance. Also this scale was translated into Italian using the backward translation technique. According to the assessment procedure adopted for the PBS-ST scale and the SEQ, we used the trait-like instructions and the same rating scales for intensity, frequency, and perceived impact dimensions.

#### The Sport Performance Psychological Inventory (IPPS-48)

A 48-item inventory [53] was developed in Italian language (Inventario Psicologico della Prestazione Sportiva; IPPS-48) to measure a range of mental skills and psychological strategies used by athletes in competition and during practice. The items pertain to eight factors, which are further included into cognitive and emotion higher-order factors. For the purposes of the current study, we administered the items included in the emotion higher-order factor only. The emotion higher-order factor comprises self-confidence (e.g., I am confident in my competitive abilities), emotional arousal control (e.g., I am able to relax and control tension when needed), worry (e.g., I feel panicked before competition), and concentration disruption (e.g., Attention wanders while competing). Athletes are asked to think about each item in terms of how frequently they have experienced the situations and the feelings described. Items are rated on a 6-point Likert type, frequency scale ranging from 1 (*never*) to 6 (*always*). CFA showed that the IPPS-48 possesses a clear factorial structure, good reliability, and the ability to distinguish among athletes of different competitive levels [53].

#### Procedure

All measures were administered following the procedure set out in Study 1 (i.e., institutional approval and administration of questionnaires). Athletes were asked to rate intensity, frequency, and perceived impact of items of the PBS-ST scale, SEQ, and PANAS referring to how they usually feel in the hour before an important competition. The items of the IPPS-48 were rated in the frequency dimension only, because of the structure of the item content (e.g., “feeling panicked” entails a high intensity of the experience) and the implicit impact on performance.

#### Data Analysis

Following Study 1, ESEM and CFA were performed to assess the factorial validity of the intensity, frequency, and perceived impact dimensions of the 15-item PBS-ST scale. We also derived descriptive statistics, Pearson product-moment correlation coefficients, reliability alpha values, and composite reliability values of the latent variables.

To analyze the invariance of the scale across the two study samples, we conducted multi-group CFAs with increasing parameter constraints one at a time. According to Brown [54], in CFA it is preferable to have balanced groups in terms of size to attain reliable and readily interpretable results. Therefore, we selected a number of participants from Study 1 based on age, gender, sport type, and sport experience who approximately matched the related demographic features of the sample in Study 2. The final sample included 229 participants from Study 1 (161 men and 68 women, 94 involved in individual and 135 in team sports), and 249 participants from Study 2 (181 men and 68 women, 87 involved in individual and 162 in team sports). After having determined a separate baseline model for each group, several increasingly stringent models were assessed to test measurement and structural invariance [42, 55]. The sequence of models tested for measurement invariance involved four different levels: configural (i.e., same number of factors and factor loading pattern across groups), weak metric (i.e., equality of the factor loadings), strong metric (i.e., equality of the factor loadings and intercepts), and strict metric (i.e., equality of error variance and covariance). Testing structural invariance entailed three steps: factor variance (i.e., equality of variance of factor scores), factor covariance (i.e., equality of covariance of factor scores), and factor mean (i.e., equality of latent means). The configural model provided a baseline value against which the subsequently specified models were compared. At each testing step, we used the likelihood ratio test for model comparison based on the Satorra-Bentler scaled chi-square difference (ΔS-B *χ*^2^) between models. We also computed the difference in CFI between models. A difference less than or equal to .01 between nested models was considered as a criterion of invariance [56].

Invariance across gender and sport type (individual vs. team) was also examined for each dimension (i.e., intensity, frequency, and functional impact) using multiple indicator, multiple cause (MIMIC) models, also called CFA with covariates [54]. We used MIMIC modeling instead of multi-group CFA because of the relatively unbalanced sample size by gender and sport (i.e., smaller number of women and athletes of individual sports in the sample compared to men and athletes of team sports). Gender and sport type covariates were dummy coded to represent group membership (i.e., woman = 0, man = 1; and individual sport = 0, team sport = 1, respectively). Then, the latent variables and indicators were regressed onto the covariates. Unlike multi-group CFA, MIMIC modeling can only test measurement invariance (indicator intercepts) and population heterogeneity (factor means). Although less flexible, MIMIC models allow more robust and parsimonious comparisons because there are fewer freely estimated parameters in the analysis of a single covariance matrix and less restrictive sample size.

After having determined the invariance of the scale across the two study samples, we conducted ESEM and CFA on the whole data from both samples (Study 1 and 2) to assess the overall measurement model, which includes intensity, frequency, and perceived impact dimensions. Finally, we ascertained the factorial validity of the intensity, frequency, and perceived impact dimensions of the SEQ and the PANAS, and the frequency dimension of the IPPS-48 before examining the associations with the PBS-ST scale to determine concurrent validity.

#### Results

In the data screening procedure, two multivariate outliers were identified using Mahalanobis’ distance (*p* < .001) and then removed. ESEM and CFA results for the PBS-ST scale are reported in Table 4, while descriptive statistics, correlation coefficients, alpha values, and composite reliability values are contained in Table 5. Fig 1 shows standardized factor loadings, error variances, and correlations between latent constructs. Both ESEM and CFA yielded satisfactory fit indices on the 15-item PBS-ST scale, thereby confirming the tenability of the 2-factor, 15-item solution reached in Study 1.

**Table 4:** Fit indices for the 15-item, 2-factor models of the intensity, frequency, and perceived impact dimensions of the PBS-ST scale from Study 2. Note: ESEM = Exploratory Structural Equation Modeling, CFA = Confirmatory Factor Analysis, *χ*^2^(*df*) = chi-square (degrees of freedom), CFI = comparative fit index, TLI = Tucker Lewis fit index, RMSEA = root mean square error of approximation, SRMR = standardized root mean square residual, AIC = Akaike’s Information Criterion.

Dimension	Model	*χ*^2^(*df*)	*χ*^2^/*df*	CFI	TLI	RMSEA (90% CI)	SRMR	AIC
**Intensity**	ESEM	133.196 (76)	1.753	.940	.917	.054 (.038–.069)	.040	9088.164
	CFA	146.409 (89)	1.645	.942	.931	.050 (.035–.064)	.051	9080.212
**Frequency**	ESEM	118.563 (76)	1.560	.950	.930	.046 (.029–.062)	.040	9429.469
	CFA	151.114 (89)	1.698	.929	.917	.052 (.037–.066)	.063	9443.319
**Perceived impact**	ESEM	130.414 (76)	1.716	.946	.925	.052 (.037–.068)	.038	11816.758
	CFA	151.747 (89)	1.705	.944	.934	.052 (.038–.066)	.052	11812.744

**Table 5:** Means, standard deviations, and Pearson-product moment correlation matrix for the latent variables from Study 2. Note: Reliability alphas (left side) and composite reliability values (right side) appear in the diagonal.

Latent variables	(1)	(2)	(3)	(4)	(5)	(6)
**(1) Functional intensity**	(.828, .830)					
**(2) Dysfunctional intensity**	-.332	(.809, .813)				
**(3) Functional frequency**	.766	-.292	(.819, .816)			
**(4) Dysfunctional frequency**	-.429	.973	-.230	(.804, .809)		
**(5) Functional perceived impact**	.527	-.470	.503	-.368	(.857, .860)	
**(6) Dysfunctional perceived impact**	-.222	.151	-.201	.142	-.367	(.819, .824)
**Means**	2.62	0.87	2.73	1.22	1.69	-0.99
**Standard deviations**	0.58	0.50	0.58	0.62	0.71	0.94

Multi-group CFAs and MIMIC analyses were then conducted to assess the invariance of the scale across the two study samples. Results are shown in Table 6. The configural multiple-sample CFA model fitted the data adequately, confirming that the PBS-ST scale had the same factor structure for both study groups. The ΔS-B *χ*^2^ tests between the configural and all other nested models were not significant. In addition, the difference of the CFI value between the configural and other models was equal or less than .01. These findings provided evidence of substantial measurement and structural invariance of the three dimensions across samples. MIMIC results also indicated measurement equivalence on the three dimensions for gender and sport type. Inclusion of the gender or sport type covariates did not alter the factor structure or indicated differences in item responses (all modification indices < 4.0).

**Table 6:** Fit indices for multi-group confirmatory factor analyses of the intensity, frequency, and perceived impact dimensions of the PBS-ST scale. Note: *χ*^2^(*df*) = chi-square (degrees of freedom), *χ*^2^/*df* = chi-square/degrees of freedom, CFI = comparative fit index, TLI = Tucker Lewis fit index, RMSEA = root mean square error of approximation, SRMR = standardized root mean square residual, ΔS-B *χ*^2^ (Δ*df*) = Satorra-Bentler scaled chi-square difference test (degrees of freedom difference).

Dimension	Model	*χ*^2^(*df*)	*χ*^2^/*df*	CFI	TLI	RMSEA (90% CI)	SRMR	ΔS-B *χ*^2^ (Δ*df*)	*p* value
**Intensity**	Configural	263.711 (178)	1.482	.951	.942	.045 (.033–.056)	.053		
	Weak metric	277.337 (191)	1.452	.951	.946	.043 (.032–.054)	.058	13.205 (13)	.432
	Strong metric	285.062 (206)	1.384	.955	.954	.039 (.027–.050)	.059	20.449 (28)	.848
	Strict metric	301.537 (221)	1.364	.954	.956	.039 (.027–.050)	.062	37.878 (43)	.693
	Factor variance	285.583 (208)	1.373	.956	.955	.040 (.027–.050)	.059	20.684 (30)	.898
	Factor covariance	285.448 (207)	1.379	.955	.955	.040 (.028–.051)	.059	20.848 (29)	.865
	Factor mean	284.195 (204)	1.393	.954	.953	.041 (.029–.051)	.058	19.637 (26)	.808
	MIMIC—Gender	203.125 (102)	1.991	.941	.931	.046 (.036–.055)	.043		
	MIMIC—Sport	210.015 (102)	2.059	.938	.927	.047 (.038–.056)	.043		
**Frequency**	Configural	244.160 (178)	1.372	.957	.949	.039 (.026–.051)	.057		
	Weak metric	252.988 (191)	1.325	.959	.955	.037 (.023–.048)	.060	8.749 (13)	.792
	Strong metric	269.214 (206)	1.307	.958	.958	.036 (.022–.047)	.062	24.363 (28)	.662
	Strict metric	278.015 (221)	1.258	.963	.964	.033 (.019–.044)	.063	33.827 (43)	.840
	Factor variance	271.319 (208)	1.304	.958	.958	.036 (.022–.047)	.065	26.350 (30)	.657
	Factor covariance	269.480 (207)	1.302	.959	.958	.036 (.022–.047)	.062	24.554 (29)	.701
	Factor mean	266.993 (204)	1.309	.959	.957	.036 (.023–.047)	.061	22.213 (26)	.677
	MIMIC—Gender	186.318 (102)	1.827	.944	.934	.042 (.032–.051)	.048		
	MIMIC—Sport	180.192 (102)	1.767	.948	.939	.040 (.030–.050))	.047		
**Perceived impact**	Configural	285.344 (178)	1.603	.935	.923	.050 (.039–.061)	.059		
	Weak metric	312.592 (191)	1.637	.926	.919	.052 (.041–.062)	.070	22.204 (13)	.052
	Strong metric	319.832 (206)	1.553	.931	.929	.048 (.038–.058)	.070	33.215 (28)	.228
	Strict metric	343.561 (221)	1.555	.926	.929	.048 (.038–.058)	.073	59.161 (43)	.051
	Factor variance	323.335 (208)	1.554	.930	.929	.048 (.038–.058)	.079	36.918 (30)	.180
	Factor covariance	320.278 (207)	1.547	.931	.930	.048 (.037–.058)	.070	33.471 (29)	.259
	Factor mean	319.321 (204)	1.565	.930	.928	.049 (.038–.059)	.070	33.041 (26)	.161
	MIMIC—Gender	204.326 (102)	2.003	.930	.917	.046 (.037–.055)	.046		
	MIMIC—Sport	201.047 (102)	1.971	.932	.920	.045 (.036–.054)	.046		

To assess the overall measurement model, we performed ESEM and CFA on data from both samples (*N* = 589). While CFA analysis yielded non convergence, ESEM analysis resulted in a convergent solution. Yet, the model showed a poor fit to the data, *χ*^2^/df = 3.058, CFI = .706, TLI = .635, RMSEA = .066 (90% CI = .063–0.068), SRMR = .044. A review of modification indices indicated the presence of large residual covariance values between items of the intensity dimension with the respective items of the frequency dimension. This result most likely reflected the high degree of overlap between the item scores of intensity and frequency dimensions also shown in the high correlation values (Tables 3 and 5). In light of this evidence [42], we judged sound to respecify the model to include correlated residuals of items of the two dimensions. The respecified model yielded acceptable fit to the data, *χ*^2^/df = 1.647, CFI = .943, TLI = .919, RMSEA = .037 (90% CI = .033–0.041), SRMR = .030.

Before examining concurrent validity, we ascertained the factorial validity of measures. CFA and reliability indices on the intensity, frequency, and perceived impact dimensions of the SEQ and the PANAS, and the frequency dimension of the IPPS-48 provided evidence of acceptable factorial validity and reliability of the measures (see Table 7). Specifically, we found support for the hypothesized 5-factor structure of the SEQ and the 4-factor structure of the IPPS-48. The 2-factor structure of the PANAS was also supported after specification of correlated residual terms of two items on the Positive Affect subscale (alert and attentive) and four items on the Negative Affect subscale (nervous and jittery, afraid and scared). Aligned with Nicolas et al. [51] contentions, the procedure of including correlated error terms among redundant items of a same affect category is justified because the Watson et al.’s [49] scale used in the current study did not incorporate the content categories originally proposed by Zevon and Tellegen [57] into the factor structure of the PANAS. Thus, theoretically meaningful latent variables may have been erroneously omitted from the original model [51].

**Table 7:** Confirmatory factor analysis fit indices, reliability alpha range, and composite reliability range of the SEQ, the PANAS, and the IPPS-48 dimensions from Study 2. Note: *χ*^2^(*df*) = chi-square (degrees of freedom), CFI = comparative fit index, TLI = Tucker Lewis fit index, RMSEA = root mean square error of approximation, SRMR = standardized root mean square residual. ^1^Two correlated errors on the Positive Affect subscale, and four correlated errors on the Negative Affect subscale of the PANAS.

Instrument	Dimension	*χ*^2^(*df*)	*χ*^2^/*df*	CFI	TLI	RMSEA (90% CI)	SRMR	Alpha	Composite Reliability
**SEQ** **(5 factors)**	Intensity	311.962 (199)	1.568	.930	.919	.047 (.037–.056)	.060	.741–.863	.742–.864
	Frequency	300.075 (199)	1.508	.939	.930	.044 (.034–.054)	.053	.713–.858	.709–.860
	Perceived impact	336.433 (199)	1.691	.923	.910	.052 (.042–.061)	.064	.727–.863	.724–.863
**PANAS**^1^ **(2 factors)**	Intensity	294.361 (166)	1.773	.909	.894	.056 (.045–066)	.065	.822–.881	.813–.880
	Frequency	270.732 (166)	1.631	.919	.905	.050 (.040–.061)	.064	.806–.859	.797–.859
	Perceived impact	253.967 (166)	1.530	.942	.932	.046 (.035–.057)	.053	.876–.886	.873–.886
**IPPS-48** **(4 factors)**	Frequency	380.125 (246)	1.545	.950	.944	.046 (.037–.055)	.065	.756–.916	.773–.916

Concurrent validity of the PBS-ST scale was examined via latent factor correlations between the scale and the criterion-related measures. We employed the indications provided by Zhu [58] to guide the interpretations of the effect size of correlation coefficients. Correlations between the intensity, frequency, and perceived impact dimensions of the two PBS-ST subscales (i.e., functional and dysfunctional) with the five SEQ subscales (i.e., anger, anxiety, dejection, excitement, and happiness), the two PANAS subscales (i.e., positive and negative affect), and the four IPPS-48 subscales (i.e., self-confidence, emotional arousal control, worry, and concentration disruption) were low, ranging from -.138 to .283 (SEQ), from -.131 to .285 (PANAS), and from -.198 to .238 (IPPS-48). These results suggest that the PBS-ST scale taps unique constructs.

## Discussion

The purpose of the study was to evaluate the validity and reliability of the intensity, frequency, and perceived impact dimensions of the Italian version of the PBS-ST scale. The items of the scale are those proposed for an individualized profiling of high-level athletes [8]. Although non-standardized scales measuring athletes’ psychobiosocial states have actually been used for research and applied purposes in sport [28, 29] and physical education [24] settings, the validity and reliability of these scales was not previously established.

At a group level, participants selected all adjectives representing the eight modalities of a psychobiosocial state (i.e., affective, cognitive, motivational, volitional, bodily, motor-behavioral, operational, and communicative), thus supporting the relevance of the descriptors and the contention that athletes’ descriptions of their states reflect emotion and non-emotion content [21, 59]. At a descriptive level, mean intensity and frequency scores of items in the functional subscale were generally larger than scores of items in the dysfunctional subscale (Table 1). As expected, functional items were perceived as helpful for performance, while dysfunctional items were perceived as harmful, with the exception of three items pertaining to Anxiety(+), Pleasant affective(-), and Communicative(-) modalities that showed reverse effects.

Adequate factorial validity was observed on all dimensions for a 2-factor solution comprising 15 items, 8 functional and 7 dysfunctional. ESEM and CFA on the initial 20-item scale [8] did not provide acceptable fit to the data. Therefore, on a first step two items were discarded because they did not load on the expected factor. According to the IZOF conceptualization [2, 9], these items are purported to measure an unpleasant/functional state [i.e., Anxiety(+)] and an pleasant/dysfunctional state [i.e., Pleasant affective(-) modality]. In the IZOF model, indeed, emotion content is construed based on the 2 × 2 interplay between hedonic valence (i.e., pleasant or unpleasant experience) and functionality (functional or dysfunctional effects on performance). This interaction leads to four global emotion content categories: (a) pleasant-functional, (b) unpleasant-functional, (c) pleasant-dysfunctional, and (d) unpleasant-dysfunctional. It is assumed that pleasant-functional states (e.g., feeling enthusiastic and confident) help the performer generate and use the energy to sustain effort and coordination for task execution, while unpleasant-functional states (e.g., feeling nervous and dissatisfied) mainly serve to energize behavior toward task accomplishment. Furthermore, pleasant-dysfunctional states (e.g., feeling complacent and satisfied) are contended to reflect a lack of energy or ineffective resource recruitment and utilization, while unpleasant-dysfunctional states (e.g., feeling worried and apprehensive) would determine a waste of energy by diverting individual’s resources toward task-irrelevant cues. In this conceptual framework, the athletes’ interpretation of their states is crucial for the development of knowledge and beliefs about their performance. In particular, athletes’ interpret their conditions as pleasant or unpleasant, and functional or dysfunctional for performance depending not only on emotion intensity, but also on their knowledge, attitudes, preferences, or rejections of the experiences. These *meta-experiences* result from individuals’ spontaneous or deliberate reflection on conditions leading to success or failure, which consequently contribute to knowledge and beliefs with respect to their own experiences [59–61]. For instance, an athlete may perceive that high anxiety benefits competitive performance because specific symptoms, such as increased heart rate, are helpful in energizing behavior and directing attention to the task. On the other hand, feeling complacent and satisfied before competition can lead to ineffective investment of energy and unfocused attention, and therefore be perceived by the athlete as harmful. This knowledge when repeatedly confirmed leads to a positive attitude toward anxiety and a negative attitude toward complacency, which are then interpreted as indicators of readiness for competition or lack of energy and focus.

Study findings of idiographic assessments of psychobiosocial states with the same items of the PBS-ST scale support the above contentions [8]. In contrast to the results from an individualized assessment approach, the group-oriented results in our study do not lend support to the use of the Anxiety(+) and Pleasant affective(-) modalities in a nomothetic format as indicators of functional and dysfunctional states, respectively. As previously observed, Anxiety(+) and Pleasant affective(-) modalities showed opposite effects than those expected. This may be due to the athletes’ unstructured meta-experiences and the attribution of emotion effects based on hedonic valence according to a common belief that unpleasant states are always harmful for performance and that pleasant states are always helpful. The Communicative(-) modality also showed opposite effects than those expected, in that mean scores of perceived impact were positive rather than negative. Furthermore, the Communicative(+) modality loaded poorly in the expected factor, and therefore both communicative modalities were discarded. According to accounts of individual experiences [8], communicative processes are very idiosyncratic. Some athletes withdraw from others to attain an optimal attention focus, avoid distractions, and cope with competitive stress, whereas other athletes seek support from the coach and peers and thus display communicative and social behavior. Therefore, “communicative, outgoing, sociable, connected” and “uncommunicative, withdrawn, alone, disconnected” adjectives in the PBS-ST scale likely reflect different precompetitive behaviors that can be regarded as helpful or harmful depending on the individual.

As a final step in factor analysis, we also decided to remove Anger(-) modality from the PBS-ST scale to avoid cross loadings between latent variables, and therefore to keep a clear distinction between functional and dysfunctional factors. Adjectives of the Anger(+) modality (i.e., fighting spirit, fierce, aggressive) were included in the functional subscale in line with findings of previous research indicating that anger can play an important role in the generation or mobilization of energy [21, 62, 63]. In collision and combat sports in particular, such as rugby and karate, athletes can experience properly harnessed anger symptoms as necessary to increase effort toward task achievement and outperform the opponent [64–66]. In this perspective, also adjectives purported to represent the Anger(-) modality (i.e., furious, resentful, irritated, annoyed), can likely be interpreted as helpful rather than harmful, thereby accounting for the cross loadings observed between latent constructs.

The 2-factor, 15-item PBS-ST scale showed adequate factorial validity on the three dimensions (i.e., intensity, frequency, and perceived impact). Notably, intensity and frequency of latent dimensions were highly related in both Study 1 (functional intensity and frequency, *r* = .828; dysfunctional intensity and frequency, *r* = .957; see Table 3) and Study 2 (functional intensity and frequency, *r* = .766; dysfunctional intensity and frequency, *r* = .973; see Table 5). These high correlation coefficients may have resulted from the trait-like format of the scale whereby participants were asked to recall how they usually felt before competition. They may have found difficult to distinguish intensity from frequency of recalled psychobiosocial states. An alternative explanation may lie in the intensity and frequency of psychobiosocial states having a similar pattern just prior to competition. In either case, in future studies researchers may decide to use the PBS-ST scale in a more parsimonious manner by measuring either intensity or frequency dimensions. In contrast, the relation of intensity and frequency dimensions to the perceived impact ranged from very low to moderate, and therefore assessing perceived impact could contribute to our understanding of the individual’s experience. However, further research is needed to investigate the trend of psychobiosocial states over time, in line with research on anxiety that has demonstrated different pattern of intensity and frequency of anxiety symptoms in the time (e.g., a week) leading up to competition [32, 67]. Of note, Thomas, Picknell, and Hanton [68] compared performers’ actual and recalled responses to the Competitive State Anxiety Inventory-2 [5] at precompetition and postcompetition intervals. Memory of the frequency of their competitive anxiety symptoms was generally more reliable than memory of the intensity, and athletes were more attuned to the frequency rather than the intensity. This evidence led Thomas et al. [68] to argue in favor of the concept that the awareness of the frequency of symptoms may act as a precursor for increasing anxiety levels, and that frequency may reflect experienced symptoms more accurately when recalling emotional accounts. Accordingly, they recommended practitioners to consider frequency in addition to intensity to help performers cope more effectively with anxiety in the time preceding competition.

A second purpose of the study was to examine invariance of the PBS-ST scale across independent samples, gender, and type of sport, and to assess the concurrent validity of the PBS-ST scale. Findings provided support for substantial measurement and structural invariance on the three dimensions across samples. Measurement equivalence was also found on the three dimensions for gender and sport type. These results indicate that the PBS-ST scale can be used in the assessment of psychobiosocial states, which allows for unbiased comparison of scores across samples, male and female athletes, and individual and team sports. Concurrent validity results of the PBS-ST scale showed low associations with sport specific emotions (i.e., anger, anxiety, dejection, excitement, and happiness), sport-specific and emotion-related constructs (i.e., self-confidence, emotional arousal control, worry, and concentration disruption), and a global measure of positive and negative affect. The low relationships suggest that the PBS-ST scale gauges athletes’ functional and dysfunctional states not assessed on the other scales administered in this study. Grounded in the IZOF model [2, 9], the items included in the PBS-ST scale have been found relevant in tapping the athletes’ holistic experience across a wide range of sports [27].

In conclusion, our study offers initial validity evidence for a sport-specific tool to measure psychobiosocial states. The 2-factor structure and the internal consistency reliability have been confirmed, and the concurrent validity suggests that the scale gauges unique constructs. Future research should determine the extent to which the scale validity generalizes across samples of different cultures, age, competitive level, and sport experience. Furthermore, the instrument was used to measure trait-like aspects of psychobiosocial states based on athletes’ retrospective reports of how they typically respond. Future studies can employ a situational (state-like) version of the scale using the timeframe of right now, and examine psychobiosocial states prior to, during, or after performance, or after interventions aimed at improving individual’s conditions leading to best achievements [69–71]. Criterion validity of the scale should be also determined in comparison with additional subjective measures of emotions, objective measures of individual states, such as behavioral, biological, and neural markers [28, 72, 73], and performance criteria [29]. Overall, our results encourage the use of the PBS-ST scale and, more generally, the assessment of psychobiosocial states in the sport setting.

## Supporting Information

S1 Appendix The Psychobiosocial Items of the PBS-ST scale and the Corresponding Italian Translation. (PDF)
