# Supplementary material for: Measuring Psychobiosocial States in Sport: Initial Validation of a Trait Measure
Source: PLoS One. 2016 Dec 1;11(12):e0167448. doi: 10.1371/journal.pone.0167448 (PMC5131932; doi:10.1371/journal.pone.0167448)
Supplement: S1 Appendix — (PDF) [file pone.0167448.s001.pdf]

S1 Appendix

The Psychobiosocial Items of the PBS-ST scale and the Corresponding Italian Translation

Items	Modality
1. Enthusiastic, confident, carefree, joyful<br>*Entusiasta, fiducioso, tranquillo, felice, gioioso*	Pleasant affective(+)
2. Fighting spirit, fierce, aggressive<br>*Combattivo, grintoso, aggressivo*	Anger(+)
3. Relaxed-, coordinated-, powerful-, effortless-movement<br>*Movimento attivo, coordinato, dinamico, fluido*	Motor-behavioral(+)
4. Distracted, overloaded, doubtful, confused<br>*Distratto, deconcentrato, dubbioso, confuso*	Cognitive(-)
5. Effective-, skillful-, reliable-, consistent-task execution<br>*Prestazione efficace, abile, sicura, costante*	Operational(+)
6. (Uncommunicative, withdrawn, alone, disconnected)<br>*(Chiuso, riservato, non socievole, isolato)*	Communicative(-)
7. (Nervous, restless, discontented, dissatisfied)<br>*(Nervoso, irrequieto, scontento, insoddisfatto)*	Anxiety(+)
8. Vigorous, energetic, physically-charged<br>*Fisicamente vigoroso, pieno di energia, carico*	Bodily-somatic(+)
9. Sluggish, clumsy, uncoordinated, powerless-movement<br>*Movimento debole, goffo, scoordinato, fiacco*	Motor-behavioral(-)
10. Alert, focused, attentive<br>*Vigile, concentrato, attento*	Cognitive(+)
11. Unmotivated, uninterested, uncommitted<br>*Demotivato, disinteressato, disimpegnato*	Motivational(-)
12. (Overjoyed, complacent, pleased, satisfied)<br>*(Allegro, compiaciuto, appagato, soddisfatto)*	Pleasant affective(-)
13. Ineffective-, unskillful-, unreliable-, inconsistent-task execution<br>*Prestazione inefficace, scadente, incerta, instabile*	Operational(-)
14. (Communicative, outgoing, sociable, connected)<br>*(Comunicativo, espansivo, socievole, cooperativo)*	Communicative(+)
15. Purposeful, determined, persistent, decisive<br>*Risoluto, determinato, tenace, perseverante, deciso*	Volitional(+)
16. Worried, apprehensive, concerned, troubled<br>*Preoccupato, angosciato, scoraggiato, turbato*	Anxiety(-)
17. Motivated, committed, inspired<br>*Motivato, coinvolto, interessato*	Motivational(+)
18. Physically-tense, jittery, tired, exhausted<br>*Fisicamente teso, nervoso, affaticato, esausto*	Bodily-somatic(-)
19. (Furious, resentful, irritated, annoyed)<br>*(Furioso, risentito, rabbioso, astioso, irritato, infastidito)*	Anger(-)
20. Unwilling, undetermined, indecisive<br>*Indeciso, incerto, esitante, rinunciatario, incostante*	Volitional(-)

Note. Items 6, 7, 12, 14, 19 are in parentheses because they were not included in the final 15-item PBS-ST scale. (+) = item categorized as functional; (-) = item categorized as dysfunctional.
